# Outcomes after a first acute myocardial infarction in patients with or without congenital heart disease

**DOI:** 10.1093/eurheartj/ehag216

**Published:** 2026-05-11

**Authors:** Love Holmgren, Sacharias von Koch, Pontus Andell, Peder Sörensson, Christina Christersson, Aleksandra Trzebiatowska-Krzynska, Maria Fedchenko, Zacharias Mandelanakis, Bengt Johansson, Moman A Mohammad, Joanna Hlebowicz

**Affiliations:** Department of Cardiology, Clinical Sciences, Lund University, Skåne University Hospital, Entrance Floor, Lund 221 85, Sweden; Department of Cardiology, Clinical Sciences, Lund University, Skåne University Hospital, Entrance Floor, Lund 221 85, Sweden; Department of Physiology and Pharmacology, Karolinska Institutet, and ME Cardiology, Karolinska University Hospital, Stockholm, Sweden; Department of Physiology and Pharmacology, Karolinska Institutet, and ME Cardiology, Karolinska University Hospital, Stockholm, Sweden; Department of Medical Sciences, Cardiology, Uppsala University, Uppsala, Sweden; Department of Cardiology and Department of Medicine and Health Sciences, Linköping University, Linköping, Sweden; Department of Molecular and Clinical Medicine, Institute of Medicine, Sahlgrenska Academy, University of Gothenburg, Gothenburg, Sweden; Department of Molecular and Clinical Medicine, Institute of Medicine, Sahlgrenska Academy, University of Gothenburg, Gothenburg, Sweden; Department of Diagnostics and Intervention, Umeå University, Umeå, Sweden; Department of Cardiology, Clinical Sciences, Lund University, Skåne University Hospital, Entrance Floor, Lund 221 85, Sweden; Department of Cardiology, Clinical Sciences, Lund University, Skåne University Hospital, Entrance Floor, Lund 221 85, Sweden

**Keywords:** Acute myocardial infarction, Mortality, Adult congenital heart disease, Atherosclerosis

## Abstract

**Background and Aims:**

The life expectancy of adult patients with congenital heart disease (ACHD) has improved, thus shifting the research focus towards age-related comorbidities to continue to improve patient outcomes. This study aimed to investigate all-cause mortality and recurrent acute myocardial infarction (AMI) in adults with and without congenital heart disease.

**Methods:**

A nationwide case control study was conducted between 2000 and 2022. Patients with ACHD (*n* = 214) and controls (*n* = 275 377) who experienced their first AMI were identified. Of these, each patient (*n* = 213) with ACHD was matched with 10 controls (*n* = 2092) based on age, sex, hypertension, diabetes, hyperlipidaemia, and history of percutaneous coronary intervention or coronary artery bypass grafting. Mortality and recurrent AMI were assessed using unadjusted and adjusted Cox regression for matching and other clinical covariates.

**Results:**

Patients with ACHD were younger (58 ± 14 years) than controls (70 ± 12 years) before the matching (*P* < .001). The mean follow-up time was 6.5 and 7.3 years for patients with ACHD and controls, respectively. There was no significant difference in mortality or recurrent AMI rates at 1 year between patients with ACHD and matched controls. The mortality rate was higher in ACHD at 10 years of follow-up (hazard ratio 1.4, 95% confidence interval 1.0–1.9) but did not remain after adjustment.

**Conclusions:**

This study suggests that survival rates and the incidence of recurrent AMI in ACHD patients are similar to those of controls. Since patients with ACHD share similar cardiovascular risk factors as the general population, promoting healthy lifestyles and proactive risk management is crucial to mitigate acquired heart disease.

## Structured Graphical Abstract

**Structured Graphical Abstract:**
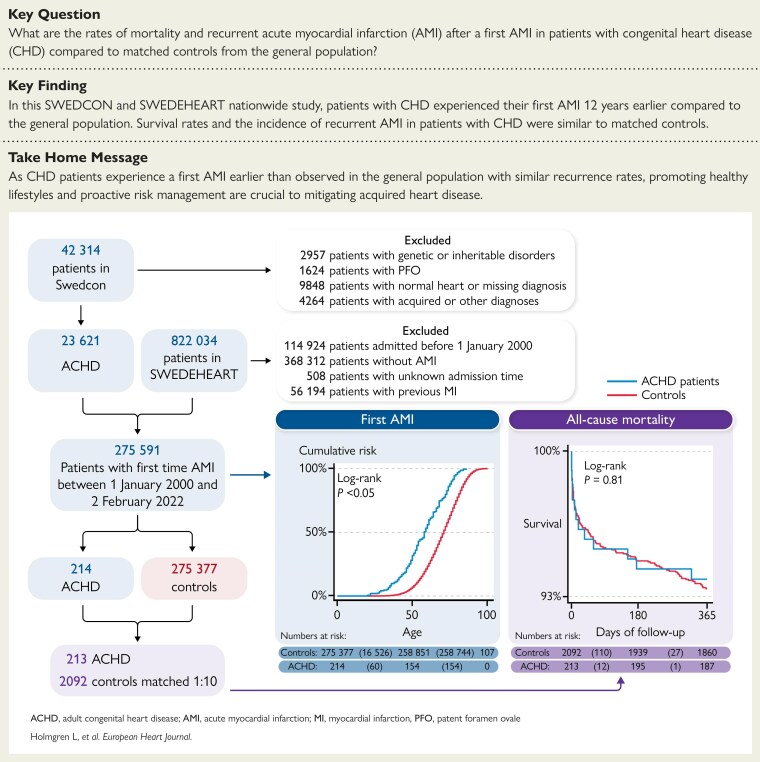
Flowchart showing the inclusion of all patients aged ≥18 years who had their first-time acute myocardial infarction (AMI) recorded in SWEDEHEART between 1 January 2000 and 2 February 2022. Cumulative risk of first AMI in 214 patients with adult congenital heart disease (ACHD) and 257,377 controls (Log rank < 0.05). Kaplan-Meier survival graph of mortality in 213 patients with ACHD and 2,092 matched controls who had an AMI between 2000 and 2022 (Log rank p = 0.81).

**See the editorial comment for this article ‘Adding insult to injury: when an adult with congenital heart disease has a heart attack’, by D. S. Celermajer,**  https://doi.org/10.1093/eurheartj/ehag160.

## Introduction

Thanks to medical advances over the past decades, survival rates for adults with congenital heart disease (ACHD) have increased dramatically. Recent figures indicate that 97% of children with congenital heart disease (CHD) are now expected to reach adulthood in developed countries.^1^ This paradigm shift has redirected research efforts from improving early survival towards understanding and managing the long-term complications that emerge in midlife and older age among ACHD patients. The risk that a patient with ACHD develops heart failure, stroke, arrhythmias, or other complications is increased compared to patients without CHD.^2,3^ In patients with ACHD under 42 years of age, the risk of ischaemic heart disease is estimated to be 16.5 times higher than in matched controls.^4,5^ Despite the increased risk of long-term cardiovascular events experienced by this subgroup of patients, these patients have been largely excluded from acute myocardial infarction (AMI) studies, and data on their treatment and outcomes are lacking.

The aim of this study was to evaluate all-cause mortality following first-time AMI in patients with and without ACHD, and to assess the risk of recurrent AMI using comprehensive, nationwide data from two population-based registries including all diagnosed cases of ACHD and AMI.

## Methods

We conducted a case-control study by identifying patients with CHD and related surgeries from the SWEDish national registry for CONgenital heart disease (SWEDCON). The registry is a nationwide registry created in the 1990s and includes data from preceding registries with diagnostic codes of CHD. The validity of the diagnoses and procedures has been approximated to be between 71–100%.^6^ Included patients with CHD were classified into mild, moderate, and severe CHD according to the European Society of Cardiology (ESC) guidelines using the NOMESCO Classification of Surgical Procedures and the European Pediatric Cardiac Codes (EPCC) registered in SWEDCON (*Table 1*, Supplementary data online, *Table S1*).^7^ Patients who had only a persistent foramen ovale or genetic disorders were excluded from this study. All patients with ACHD aged ≥18 years were screened for their first-time AMI recorded in the Swedish Web-system for Enhancement and Development of Evidence-based care in Heart disease Evaluated According to Recommended Therapies (SWEDEHEART), a nationwide quality of care registry. SWEDEHEART includes data on all patients in Sweden admitted to a cardiac intensive care unit as well as all patients who underwent invasive coronary angiography or percutaneous coronary intervention (PCI),^8^ recording cardiovascular risk factors, troponin levels, electrocardiogram (ECG) findings, and coronary angiograms. The inclusion period was set to 1 January 2000 until 2 February 2022 (see Supplementary data online, *Figure S1*). There were 114 924 patients admitted before 1 January 2000 who were excluded. An AMI was defined as an ST-elevation myocardial infarction (STEMI) or non-STEMI (NSTEMI), determined clinically based on the World Health Organization’s 10th version of International Classification of Diseases (ICD-10) codes I21, I21.0-I21.4, I21.4A-B, I21.4W, I21.4X and I21.9.^8^ Linkage between registries was done using the unique personal identification numbers of each Swedish citizen. Vital status was ascertained by the National Board of Health and Welfare by linking SWEDEHEART and SWEDCON to the National Population Registry. For privacy purposes, the personal identification numbers were removed before we retrieved the linked registries. All variables, excluding date of death and ACHD diagnoses, were retrieved from SWEDEHEART, and all variables, excluding ACHD diagnoses, had been recorded between 1 January 2000 and 2 February 2022.

**Table 1:** Classification of congenital heart disease (CHD) and CHD severity in patients with and without acute myocardial infarction

	All CHD (*n* = 22 830)	CHD with AMI (*n* = 214)
Mild CHD		
Isolated congenital aortic valve disease and bicuspid aortic disease	2537 (11)	17 (8)
Isolated congenital mitral valve disease	847 (4)	5 (2)
ASD, PDA or sinus venosus defect without associated abnormalities^a^	5525 (24)	81 (38)
Tricuspid valve abnormalities	158 (1)	1 (<1)
VSD without associated abnormalities^a^	3646 (16)	10 (5)
Pulmonary valve abnormalities	2107 (9)	17 (8)
Total	14 820 (64)	131 (61)
Moderate CHD		
Anomalous pulmonary venous connection (partial or total)	711 (3)	10 (5)
Anomalous coronary artery	204 (1)	6 (3)
Aortic stenosis—sub valvular or supravalvular	500 (2)	2 (1)
AVSD, partial or complete, including primum ASD	598 (3)	4 (2)
Coarctation of the aorta	2009 (9)	21 (10)
Double chambered right ventricle	5 (<1)	0
Ebstein anomaly	248 (1)	7 (3)
Peripheral pulmonary stenosis	215 (1)	0
Sinus of Valsalva aneurysm/fistula	52 (<1)	1 (<1)
Tetralogy of Fallot	911 (4)	13 (6)
Transposition of the great arteries after arterial switch operation	152 (1)	0
VSD with associated abnormalities^a^	125 (1)	1 (<1)
Parachute mitral valve, cleft leaflet	32 (<1)	0
Aortic dissection	14 (<1)	0
PDA, ASD secundum, moderate/large unrepaired with abnormalities*	122 (1)	5 (2)
Total	5898 (26)	70 (32)
Severe CHD		
CHD with secondary pulmonary hypertension (Eisenmenger syndrome)	138 (1)	1 (<1)
Double-outlet ventricle	89 (<1)	0
Univentricular heart physiology with Fontan circulation or total cavopulmonary connection	333 (1)	0
Interrupted aortic arch	146 (1)	1 (<1)
Pulmonary atresia (all forms)	279 (1)	3 (1)
Transposition of the great arteries (except arterial switch)	843 (4)	7 (3)
Truncus arteriosus	104 (<1)	0
Other complex abnormalities (crisscross heart, ventricular inversion)	185 (1)	1 (<1)
Total	2117 (9)	13 (6)

Data are presented as *n* (%).

ASD, atrial septal defect; AVSD, atrioventricular septal defect; PDA, patent ductus arteriosus; VSD, ventricular septal defect.

^a^Ventricular dysfunction or secondary pulmonary hypertension.

### Outcomes

The primary outcomes were all-cause mortality and recurrent AMI. Patients were followed until death or the end of the study period. Secondary objectives included the ECG findings, troponin levels, angiographic findings, and cardiovascular risk factors. Hypercholesterolaemia was defined as statin treatment or having a level of low-density lipoprotein (LDL) measured above 1.4 mmol/L during hospital admission. For angiographic findings, a significant stenosis was defined as a ≥50% luminal stenosis.

### Statistical analysis

Continuous variables with normal distribution were analysed with the Student’s *t*-test and presented as means with standard deviation; those without normal distribution were analysed with the Mann-Whitney U test and presented as medians with interquartile range (IQR). The Chi-squared test was used to calculate categorical variables. A matched cohort was created using propensity score matching. Logistic regression was used to calculate propensity scores of age, sex, hypertension, diabetes, hypercholesterolaemia, previous PCI, and previous coronary artery bypass grafting (CABG), as these are major determinants of mortality. A nearest neighbour approach with a no replacement method (with a calliper of 0.2) was used to match 10 controls (patients without ACHD) with one ACHD patient. Cox regression was used to assess mortality and recurrent AMI in the matched sample, with and without adjustments for matching covariates, atrial fibrillation or flutter, aspirin use, systemic ventricular ejection fraction, and smoking status. For the Cox proportional hazards model, the 11-member cluster (comprising each case and its 10 matched controls) was included as a random frailty term to account for within-cluster correlation.

Patients with missing values in the variables that were matched or adjusted for were excluded from the analysis. The results were presented as hazard ratios (HR) with 95% confidence intervals (CI). A 30-day blanking period was applied for recurrent AMI early readmissions, as follow-up visits and hospital transfers could be incorrectly interpreted as recurrent AMI due to national coding practices. Subgroup analyses were conducted to explore potential effect modification by sex, age (≥75 years), diabetes mellitus, hypertension, and employment status. Hazard ratios with 95% CIs were estimated using Cox proportional hazards models. Interaction between ACHD and each subgroup variable was included in the models, and *P*-values for interaction were calculated to assess whether the association between ACHD and all-cause mortality differed across strata. Propensity score matching was conducted in R (version 4.5.0; R Foundation for Statistical Computing, Vienna, Austria). All other analyses were performed with Stata (version 18.0; StataCorp, College Station, TX, USA). All *P*-values were two-tailed and considered statistically significant if they were less than .05.

## Results

A total of 214 patients with ACHD (see Supplementary data online, *Figure S1*, *Table 2*) and 275 377 controls with first-time AMI were included in this study. Patients with ACHD were, on average, 12 years younger than controls (58 ± 15 vs. 70 ± 12 years). The prevalence of diabetes, hypertension, hypercholesterolaemia, and smoking was similar. However, patients with ACHD had significantly lower levels of both LDL cholesterol (2.9 ± 1.0 vs 3.1 ± 1.0 mmol/L) and total cholesterol (4.8 ± 1.2 vs 5.1 ± 1.4 mmol/L) compared with the controls. A higher proportion of patients with ACHD had reduced systemic ventricular ejection fraction) before the AMI compared to controls (*Table 2*). Five percent of patients with ACHD and 1% of controls had a previous prescription of direct oral anticoagulants (DOACs) (*Table 2*). Thirty-eight of the 92 (42%) patients with atrial septal defect (ASD) without associated abnormalities had their ASD closed before their first AMI. Of these 38 patients with closed ASD, 21 (57%) had open heart surgery, and 17 (44%) had a catheter-based intervention.

**Table 2:** Baseline characteristics in patients with and without adult congenital heart disease who had a first-time acute myocardial infarction in Sweden between 2000 and 2022

	ACHD (*n* = 214)	Controls (*n* = 275 377)	*P*-value
**Age, years, mean (SD)**	58 (15)	70 (12)	<.001
**Male sex assigned at birth**	135 (63)	175 985 (64)	.80
**Diabetes mellitus**	14 (7)	23 926 (9)	.27
**Hypercholesterolaemia**	165 (77)	198 656 (72)	.11
**LDL-cholesterol, nmol/L, mean (SD)**	2.9 (1)	3.1 (1)	.013
**Total cholesterol, nmol/L, mean (SD)**	4.8 (1.2)	5.1 (1.4)	.013
**BMI, kg/m^2^, mean (SD)**	27 (5)	27 (5)	.34
**Arterial hypertension**	85 (40)	127 760 (46)	.050
**Previous CABG**	9 (4)	7199 (3)	.15
**Previous PCI**	11 (5)	7444 (3)	.028
**Previous stroke**	16 (9)	14 787 (7)	.31
**Occupation**			
** Working**	86 (48)	58 020 (26)	<.001
** Sick leave**	12 (7)	5110 (2)	
** Unemployed**	3 (2)	3363 (2)	
** Retired**	75 (42)	154 216 (70)	
** Studies/other**	4 (2)	878 (<1)	
**Smoking**			
** Never smoked**	101 (49)	114 632 (45)	.33
** Ex-smoker >1 month**	64 (31)	82 047 (32)	
** Smoker**	40 (20)	59 655 (23)	
** Systemic ventricular ejection fraction before AMI**			
** Normal (≥50%)**	169 (93)	222 439 (98)	<.001
** Mildly impaired (40–49%)**	6 (3)	2053 (1)	
** Moderately impaired (30- 39%)**	4 (2)	1088 (<1)	
** Severely impaired (<30%)**	2 (1)	599 (<1)	
**DOACs**	11 (5)	4066 (1)	<.001
**Aspirin**	169 (80)	239 151 (89)	<.001
**Warfarin**	28 (13)	15 637 (6)	<.001

Data are presented as *n* (%) unless otherwise indicated.

ACHD, adult congenital heart disease; AMI, acute myocardial infarction; BMI, body mass index. PCI, percutaneous coronary intervention. CABG, coronary artery bypass graft. DOAC, direct oral anticoagulant; SD, standard deviation; LDL, low-density lipoprotein.

Both groups had similar symptoms and levels of troponin. Right bundle branch block and pacemaker ECG were more common among patients with ACHD than in controls (*Table 3*). The distribution of STEMI and NSTEMI was similar between patients with ACHD and controls (*Table 3*). The prevalence of significant stenosis on coronary angiogram was higher in controls compared to patients with ACHD (*Table 3*). The choice of revascularization strategy was similar with 82% of controls vs 80% of patients with ACHD being treated with PCI. CABG was performed in 11% of controls and in 8% of patients with ACHD. Twelve percent of ACHD patients and 6% of controls received no revascularization therapy.

**Table 3:** Secondary outcomes in patients with and without adult congenital heart disease who had an acute myocardial infarction between 2000 and 2022

	ACHD (*n* = 214)	Controls (*n* = 275 377)	*P*-value
**Presenting symptoms**			
** Chest pain**	167 (80)	223 723 (84)	.42
** Dyspnoea**	14 (7)	15 978 (6)	
** Cardiac arrest**	4 (2)	3728 (1)	
** Other**	23 (11)	22 141 (8)	
**ECG: rhythm**			
** Sinus rhythm**	173 (82)	239 522 (88)	<.05
** Atrial fibrillation or flutter**	26 (12)	26 209 (10)	
** Other**	12 (6)	32 418 (12)	
**ECG: QRS complex**			
** Normal**	116 (56)	179 656 (67)	<.05
** Pacemaker rhythm**	7 (3)	2836 (1)	
** Left bundle branch block**	10 (5)	13 811 (5)	
** Pathological Q-waves**	14 (7)	30 765 (11)	
** Right bundle branch block**	28 (13)	9888 (4)	
** Other**	34 (16)	32 418 (12)	
**Troponin T, ng/L, mean (SD)**	23 (96)	57 (528)	.69
**Troponin I, ng/L, mean (SD)**	14 (20)	32 (325)	.69
**Diagnosis**			
** STEMI**	47 (32)	65 268 (38)	.31
** NSTEMI**	100 (68)	107 233 (62)	
**Occlusion scale**			
** 1 artery no main branch**	65 (67)	65 788 (47)	<.05
** 2 arteries no main branch**	15 (15)	38 130 (27)	
** 3 arteries no main branch**	11 (11)	25 680 (18)	
** Main branch**	3 (3)	453 (<1)	
** Main branch + 1 artery**	1 (1)	1451 (1)	
** Main branch + 2 arteries**	1 (1)	2795 (2)	
** Main branch + 3 arteries**	1 (1)	5195 (4)	
**Occluded LCA**	41 (22)	71 558 (36)	<.05
**Occluded RCA**	82 (44)	122 458 (61)	<.05

Data are presented as *n* (%).

Troponin T and troponin I are reported separately, as these biomarkers were analysed in different years and across different regions. Location and scale of significant stenosis in coronary angiograms of patients with (*n* = 103) and without ACHD (*n* = 141 802) who had an acute myocardial infraction between 2000 and 2022.

ACHD, adult congenital heart disease; ECG, electrocardiogram; LCA, left coronary artery. NSTEMI, non-ST-elevation myocardial infarction; RCA, right coronary artery; STEMI, ST-elevation myocardial infarction.

The systemic ventricular ejection fraction was depressed after the AMI in 44% of patients with ACHD and 42% of controls; the difference was not statistically significant. The proportion of patients with depressed systemic ventricular ejection fraction after AMI did not differ between patients with or without CHD (44 vs 42%, *P* = .674). Aspirin was prescribed at discharge to 80% of patients with ACHD and 89% of controls (*P* < .001). DOACs were prescribed to 7% of ACHD patients and 3% of controls (*P* < .001), while warfarin was prescribed to 13% of ACHD patients and to 6% of controls (*P* < .001). The prescription of P2Y_12_ inhibitors, statins, and antihypertensive drugs was similar in patients with and without CHD.

After matching each ACHD patient with 10 controls, 213 ACHD patients and 2092 controls were included in the analysis of mortality and recurrent AMI. In this subset, patients with ACHD had a lower body mass index, a higher prevalence of previous stroke, were less frequently smokers, had a higher prevalence of systemic ventricular dysfunction, systemic ventricular ejection fraction, and were treated more frequently with DOACs (*Table 4*). The median follow-up time was 10 years in ACHD patients (IQR 5-15) and 11 years in controls (IQR 6–16). Sixty-five (31%) patients with ACHD and 587 (28%) controls died during the follow-up. Eleven (5%) patients with ACHD and 107 (5%) controls died within 180 days. Twelve (6%) with ACHD and 130 (7%) controls died within 1 year, and 30 (28%) with ACHD and 274 (23%) controls died within 10 years. Recurrent AMI occurred in 20 (9%) patients with ACHD and 308 (15%) controls.

**Table 4:** Baseline characteristics in patients with and without adult congenital heart disease who were matched by age and sex and who had a first-time acute myocardial infarction in Sweden between 2000 and 2022

	ACHD (*n* = 213)	Controls (*n* = 2092)	*P*-value
**Age, years, mean (SD)**	58 (14)	58 (14)	.59
**Male sex assigned at birth**	134 (63)	1284 (61)	.66
**Diabetes mellitus**	14 (7)	144 (7)	.86
**Hypercholesterolaemia**	164 (77)	1653 (79)	.49
**LDL-cholesterol, nmol/L, mean (SD)**	2.9 (1)	3.2 (1)	.001
**Total cholesterol, nmol/L, mean (SD)**	4.8 (1.2)	5.2 (1.3)	<.001
**BMI, kg/m^2^, mean (SD)**	27 (5)	28 (5)	.009
**Arterial hypertension**	85 (40)	844 (40)	.90
**Previous CABG**	8 (4)	60 (3)	.47
**Previous PCI**	11 (5)	99 (5)	.78
**Previous stroke**	16 (9)	71 (4)	.008
**Occupation**			
** Working**	86 (48)	796 (48)	.47
** Sick leave**	11 (6)	94 (6)	
** Unemployed**	3 (2)	62 (4)	
** Retired**	75 (42)	678 (41)	
** Studies/other**	4 (2)	19 (1)	
**Smoking**			
** Never smoked**	100 (49)	762 (38)	<.001
** Ex-smoker >1 month**	64 (31)	524 (26)	
** Smoker**	40 (20)	714 (36)	
** Systemic ventricular ejection fraction before AMI**			
** Normal (≥50%)**	169 (93)	1732 (99)	<.001
** Mildly impaired (40–49%)**	6 (3)	10 (1)	
** Moderately impaired (30–39%)**	4 (2)	10 (1)	
** Severely impaired (<30%)**	2 (1)	1 (<1)	
**DOACs**	11 (5)	19 (1)	<.001
**Aspirin**	168 (80)	1891 (92)	<.001
**Warfarin**	28 (13)	98 (5)	<.001

Data are presented as *n* (%) unless otherwise indicated.

ACHD, adult congenital heart disease; AMI, acute myocardial infarction; BMI, body mass index. PCI, percutaneous coronary intervention; CABG, coronary artery bypass graft; DOAC, direct oral anticoagulant; LDL, low-density lipoprotein; SD, standard deviation.

Thirty-nine (30%) patients with mild CHD, 22 (31%) with moderate, and 4 (33%) patients with severe CHD died during follow-up. Among patients with STEMI, 12 (26%) ACHD patients and 82 (15%) controls died. In patients with NSTEMI, 21 (21%) ACHD and 149 (20%) controls died. In the group of patients who had an angiogram showing significant stenosis, 20 (20%) ACHD patients and 195 (17%) controls died. For those who did not have a significant stenosis, 29 (33%) patients with ACHD and 172 (27%) controls died.

Twelve (9%) patients with mild CHD, 8 (11%) with moderate, and no patients with severe ACHD had a recurrent AMI during follow-up. Among patients with STEMI, two (4%) ACHD patients and 44 (8%) controls had a recurrent AMI. In patients with NSTEMI, eight (8%) ACHD and 79 (10%) controls had a recurrent AMI. In the group of patients who had an angiogram showing significant stenosis, 10 (10%) ACHD patients and 111 (9%) controls had a recurrent AMI. For those who did not have a significant stenosis, eight (9%) patients with ACHD and 129 (20%) controls had a recurrent AMI.

**Figure 1:**
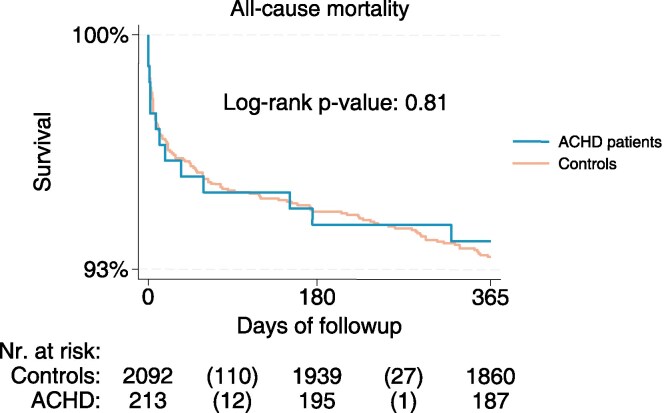
Kaplan-Meier survival graph of mortality in 213 patients with adult congenital heart disease (ACHD) and 2092 matched controls based on age, sex, hypertension, diabetes, hyperlipidaemia, and history of percutaneous coronary intervention or coronary artery bypass grafting who had an acute myocardial infarction between 2000 and 2022 (log-rank *P* = .81). Sixty-five patients with ACHD and 587 controls died during the follow-up period

**Figure 2:**
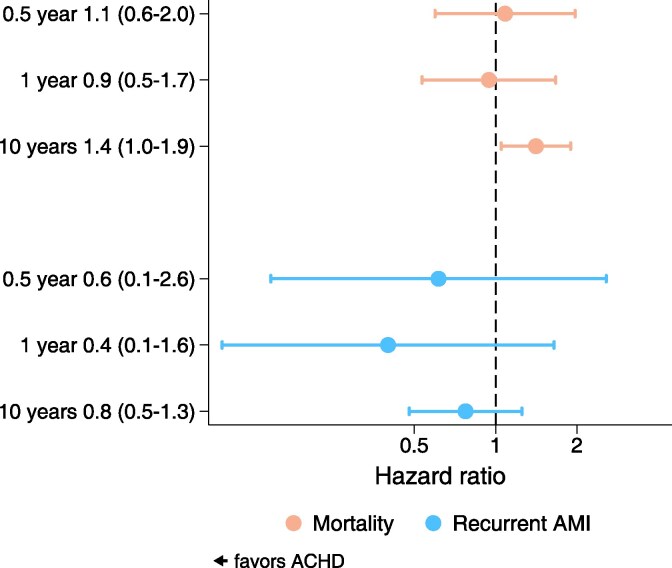
Adjusted Cox regression of mortality and recurrent acute myocardial infarction (AMI) hazard ratios (HR) at 180 days and 1 year of follow-up in 213 patients with adult congenital heart disease (ACHD) and 2092 controls who were matched 1:10 by age, sex, hypertension, diabetes, hyperlipidaemia, and history of percutaneous coronary intervention or coronary artery bypass grafting. Adjustments were made for atrial fibrillation or flutter, aspirin use, and smoking. A total of 65 patients with ACHD and 587 controls died during the follow-up period. Recurrent AMI occurred in 20 ACHD patients and 308 controls

There was no significant association between CHD and mortality at 180 days (HR 1.1, 95% CI 0.60–2.0) or 1 year (HR 0.94, 95% CI 0.53–1.7), after AMI (*Figure 1*). However, the mortality HR was higher in ACHD at 10 years of follow-up (HR 1.4, 95% CI 1.05–1.90) (*Figure 2*) (*Table 5*). The hazard rates of recurrent AMI were similar at 180 days (HR 0.61, 95% CI 0.15–2.6), 1 year (HR 0.40, 95% CI 0.10–1.6), and 10 years of follow-up (HR 0.77, 95% CI 0.48–1.3) of follow-up (*Figure 2*). The associations for mortality or recurrent AMI did not change after adjusting for atrial fibrillation or flutter, aspirin use, smoking, and reduced left ventricular ejection fraction, except the mortality rates at 10 years, which became similar after applying the adjustments (HR 1.13, 95% CI 0.75–1.70) (*Table 5*). For 1-year mortality, reduced systemic ventricular ejection fraction, current or former smoking, older age, and hypertension were all associated with an increased risk. In contrast, both aspirin use and hypercholesterolaemia showed a protective effect. For 10-year mortality, a similar pattern was observed, with an additional increased risk associated with previous CABG, type 2 diabetes mellitus, and atrial fibrillation (see Supplementary data online, *Table S2*).

**Table 5:** Cox regression showing hazard ratios of all-cause mortality and recurrent acute myocardial infarction in 213 patients with adult congenital heart disease and 2092 matched controls based on age, sex, hypertension, diabetes, hyperlipidaemia, and history of percutaneous coronary intervention or coronary artery bypass grafting

Model	All-cause mortality						Recurrent AMI					
	180 days		1 year		10 years		180 days		1 year		10 years	
	HR	95% CI	HR	95% CI	HR	95% CI	HR	95% CI	HR	95% CI	HR	95% CI
**1**	1.08	.60–1.97	.94	.53–1.67	1.41	1.05–1.90	0.61	0.15–2.57	0.40	0.10–1.64	0.77	0.48–1.25
**2**	0.61	0.23–1.61	0.52	0.21–1.27	1.13	0.75–1.70	1.04	0.23–4.79	0.61	0.14–2.65	1.06	0.63–1.79
**3**	1.09	0.60–1.99	0.94	0.53–1.66	1.45	1.08–1.94	0.60	0.14–2.53	0.39	0.09–1.59	0.74	0.46–1.19
**4**	0.58	0.23–1.47	0.52	0.21–1.25	1.25	0.85–1.84	0.96	0.20–4.75	0.62	0.14–2.75	1.06	0.63–1.79

There were four adjustment models. The first model represented crude HR. The second model adjusted for adjustments for atrial fibrillation or flutter, aspirin use, systemic ventricular ejection fraction, and smoking status. The third model adjusted for age, sex, hypertension, diabetes, hypercholesterolaemia, previous PCI, and previous CABG. The fourth model adjusted for age, sex, hypertension, diabetes, hypercholesterolaemia, previous PCI and previous CABG, atrial fibrillation or flutter, aspirin use, systemic ventricular ejection fraction, and smoking status.

AMI, acute myocardial infarction; CABG, coronary artery bypass grafting; CI, confidence interval; HR, hazard ratio; PCI, percutaneous coronary intervention.

**Figure 3:**
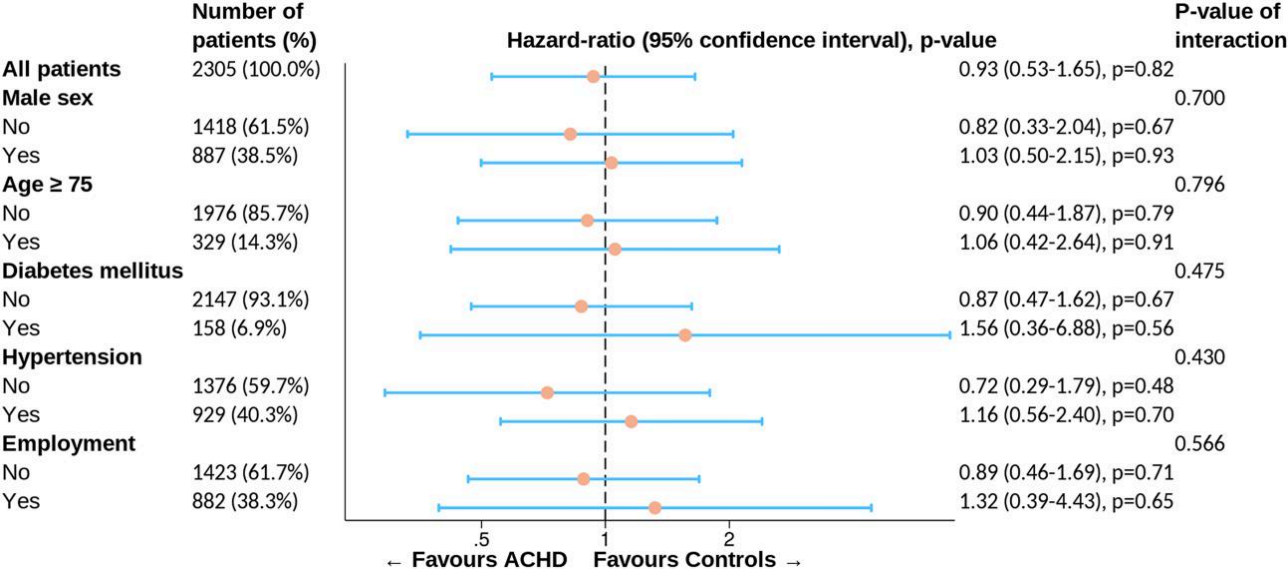
Forest plot of hazard ratios (HR) with 95% confidence intervals for all-cause mortality after first-time acute myocardial infarction (AMI) in patients with adult congenital heart disease (ACHD) compared with controls. Analyses were stratified by sex, age ≥75 years, diabetes mellitus, hypertension, and employment status. The vertical dashed line represents HR = 1 (no difference). None of the subgroups showed significant differences, and *P*-values for interaction indicated no evidence of effect modification.

Subgroup analyses were performed for sex, age ≥75 years, diabetes mellitus, hypertension, and employment status. Among these subgroups, no significant differences in all-cause mortality and no treatment-by-subgroup interaction were observed (*Figure 3*). There was no significant difference in the hazard rate of mortality between patients with mild ACHD and patients with moderate or severe ACHD at 180 days (HR 0.80, 95% CI 0.24–2.65), 1 year (HR 0.99, 95% CI 0.33–3.04), or 10 years (HR 1.11, 95% CI 0.63–1.95) of follow-up.

## Discussion

The purpose of this study was to compare mortality and recurrence after the first AMI in patients with CHD compared to matched controls from the general population, in relation to known cardiovascular risk factors and angiographically confirmed atherosclerosis. Our findings suggest that 1-year survival rates and the incidence of recurrent AMI in ACHD patients were similar to those of matched controls by demographical and clinical characteristics, including age, sex, and common cardiovascular risk factors, as well as prior coronary interventions (*Structured Graphical Abstract*).

However, ACHD patients experienced their first AMI 12 years earlier compared to the general population. This could be explained by ACHD patients with AMI being predominantly in the mild or moderate lesion group. This likely reflects the higher prevalence of these defects in the population, improved survival among patients with complex CHD in recent decades, and potential immortal time bias.^1^ The reassuring post-AMI mortality in ACHD must be interpreted in light of the cohort’s younger age distribution, which differs markedly from the general population and may potentially be explained by a historically shorter life expectancy in the ACHD group, resulting in a younger cohort compared to the general population. The initially observed higher long-term (10-year) mortality among ACHD patients disappeared after adjustment for atrial fibrillation or flutter, aspirin use, systemic ventricular ejection fraction, and smoking, suggesting that the excess risk may be explained by these comorbidities rather than ACHD itself. Known cardiovascular risk factors such as diabetes, hypercholesterolaemia, hypertension, and smoking at the time of the first AMI did not differ between ACHD patients and the general population. Interestingly, ACHD patients presented with less extensive coronary artery disease on angiography compared to the general population which could be explained by their younger age. However, given that 47% of patients with ACHD were employed at the time of presentation (compared to only 26% in the controls), early AMI in this population may carry a disproportionately greater societal impact, including loss of productivity and increased healthcare and social support needs. Fedchenko *et al*.^5^ showed that all-cause mortality after an initial AMI was slightly higher in ACHD patients compared to controls, indicating an increased risk of death following an AMI in ACHD patients relative to the control group. Similar to our findings, no difference was observed in recurrent AMI.^5^ In addition, the increased rate of the composite of recurrent AMI, heart failure, and death due to cardiovascular disease in ACHD was mainly driven by an increased risk of new-onset heart failure.^5^ Our study indicates that ACHD patients had reduced systemic ventricular function at baseline compared to controls. There was no difference in systemic ventricular ejection fraction after the first myocardial infarction among ACHD patients and controls. However, lower systemic ventricular ejection fraction after AMI, previous CABG, type 2 diabetes mellitus, atrial fibrillation, hypertension, older age, and current or former smoking were associated with an increased risk of long-term mortality, while aspirin use and hypercholesterolaemia appeared protective. Hypercholesterolaemia, likely reflecting the protective effect of statin therapy.

As we lack detailed information on the cause of death, we cannot exclude that mortality at 10 years after AMI may be driven by factors unrelated to the index AMI, which should be considered when interpreting these findings. A greater overall risk for death in the ACHD group was reported in a study from Denmark.^9^ Unfortunately, this study is not fully comparable to ours, as that study includes patients from 1977 to 1999 before the widespread use of PCI and contemporary secondary prevention and risk factor intervention, along with data from 2000 to 2012. In contrast to Fedchenko *et al*., our study utilizes clinically validated AMI diagnoses from the SWEDEHEART registry rather than administrative databases, and high accuracy and detailed CHD classification from SWEDCON, enabling more precise assessment of both cardiovascular events and CHD characteristics. Our study also addresses a key gap by examining an older ACHD population that has not been well represented in previous research. By including patients born between 1895 and 2001, we provide unique insights into contemporary management strategies and long-term prognosis in this aging ACHD cohort. Together with data on revascularization methods, drug use, and long-term follow-up in the contemporary PCI era, this underscores the novelty of our study.

The mechanisms underlying early development of ischaemic heart disease in ACHD are multifactorial. Previous surgical procedures and their associated physiological responses may play a role in the onset of ischaemia. Many individuals with ACHD experience reduced maximal oxygen uptake combined with increased oxygen demand due to volume and pressure overload that exposes them to myocardial ischaemia even with normal coronary arteries.^9^ Conventional risk factors, such as diabetes^10^ and arterial hypertension,^11–13^ may also contribute to the early development of atherosclerosis in patients with ACHD. In this study, we found that ACHD patients had lower LDL cholesterol and total cholesterol. Conventional risk factors such as diabetes and hypertension were present at similar rates in both patients with and without CHD, indicating that these factors are relevant to the development of atherosclerosis in ACHD patients as well. In an ageing cohort of ACHD patients with abnormal anatomy and physiology, the impact of established cardiovascular risk factors may be augmented. These patients are already at increased risk for systemic ventricular dysfunction, arrhythmias, and overt heart failure^2,3^ making then even more vulnerable for the effects of ischaemic heart disease.

The most common cardiac diagnoses in patients with the first AMI were isolated ASD, followed by coarctation of the aorta (CoA) (*Table 1*, Supplementary data online, *Table S1*). ASD association with coronary artery disease has been described previously.^4,14^ This is most likely because these defects are more common and patients have reached older ages. A proposed mechanism is emboli travelling through an atrial shunt and reaching the coronary arteries resulting in myocardial infarction.^9,15,16^ We found no evidence to support this in our data, suggesting that cases of embolism may have been underreported in the registry. CoA has also previously been reported to be associated with a higher risk of AMI.^4,14^ In previous studies, CoA has been linked to hypertension,^11–13^ abnormal vascular reactivity^13^ that may contribute to atherosclerosis^12^ and vascular events in individuals with this condition. Our previous research has shown that women with moderate to severe CHD have a slightly higher body weight, a greater proportion of fat mass, an elevated waist-to-hip ratio, and increased visceral adipose tissue, all indicating an unhealthy fat distribution around the waist.^17^ Abdominal overweight is traditionally recognized as a significant risk factor for developing acquired cardiovascular disease.^18^ Increased fat mass may be modifiable through interventions like strength training and dietary adjustments.^18^ The current ESC guidelines on sports and exercise recommend regular moderate exercise for individuals with CHD, shifting the focus from historical exercise restrictions to encouraging physical activity.^19^ Uncertainty about the risks and benefits of physical activity in patients with CHD has often led to a lifelong sedentary lifestyle, starting in childhood. An essential first step is to reassure patients of the safety and benefits of being active. Early risk factor screening and integration of ACHD patients into secondary prevention programmes should be emphasized, as suggested by a state-of-the-art overview of acquired heart disease in ACHD and practical guidance on prevention.^20^ To reduce cardiovascular risk, both traditional factors and maybe also non-traditional factors such as chronic inflammation and psychosocial stress, should be actively identified and addressed.^21^

We found that patients with ACHD were less likely than controls to be prescribed aspirin at discharge after the first AMI. One possible explanation is that patients with CHD were more frequently prescribed DOACs and warfarin at both admission and discharge, as atrial fibrillation and atrial flutter are commonly observed in this population, which may indicate a higher likelihood of an embolic cause of AMI. The amounts of angiotensin-converting enzyme inhibitors, beta-blockers, statins, DOACs and P2Y_12_ inhibitors were prescribed similarly in both groups.

The clinical implication of this study is that survival rates and the incidence of recurrent AMI appear to be similar in patients with and without CHD but more research is needed. Cardiologists and general practitioners should take this into account when they assess ACHD patients with AMI. Despite their higher burden of healthcare utilization, treating patients with ACHD and AMI will result in a good outcome comparable to the background population.

This study is the first to compare long-term outcomes after AMI in patients with and without CHD during the PCI era, incorporating detailed knowledge of CHD diagnoses, previous interventions, as well as comprehensive data on angiography, PCI, and CABG. A strength of the study is that there is almost no loss of follow-up for mortality. Surprisingly, a higher proportion of patients with CHD had normal coronary arteries or non-significant stenoses. This may reflect certain limitations in the study and suggests the possibility that type 2 myocardial infarctions, which are more common in the context of heart failure and arrhythmias, frequent in ACHD patients, may have been included in our study.^2,3,22^However, patients without CHD also exhibited clean or non-significant stenoses, with a prevalence of 6% compared to 19% in ACHD patients. In younger patients with ACHD presenting with chest pain, there may be a lower index of suspicion for ischaemic causes, which can delay diagnosis and appropriate management.

There are some limitations to this study. The retrospective nature of this study introduces inherent limitations, including potential misclassification, incomplete data capture, and inability to establish causality. We lack data on several known cardiovascular risk factors such as hyperlipidaemia and diabetes, for patients with ACHD who have not experienced an AMI, and this limitation restricted our ability to compare the risk of AMI in patients with ACHD who experienced AMI with those who did not. Future studies should continue to research further the mechanisms underlying the increased risk of AMI in patients with ACHD. It is especially important to conduct studies that demonstrate how we can modify interventions to reduce the risk of AMI. Investigating risk factors is crucial for early primary prevention, and it is essential to identify which CHD lesions carry the highest risk and why. Although SWEDEHEART does not explicitly differentiate between type 1 and type 2 AMI, the availability of angiographic data allows us to identify patients with significant coronary artery disease who underwent PCI, supporting the likelihood that a substantial proportion of cases represent type 1 AMI. Nevertheless, we acknowledge that type 2 AMI may have occurred, particularly in ACHD patients with heart failure or arrhythmias. Potential sources of bias such as immortal time bias, depletion of susceptible factors and selection bias should be acknowledged. Also, the absence of data on medication adherence and lifestyle factors during follow-up may have introduced residual confounding. A further limitation is that the cause of death data were not available and can therefore not be reported. Finally, we cannot completely exclude the possibility that individuals with CHD who have never been admitted to ACHD specialist care were included among the controls. However, the high coverage and validated diagnostic accuracy of SWEDCON substantially reduce this risk.

## Conclusions

Our findings suggest that survival rates and the incidence of recurrent AMI in ACHD patients is comparable to that of matched controls. Given that ACHD patients share the same cardiovascular risk factors as the general population, it is crucial to identify and address these risks early on. Lifelong exposure to cardiovascular risk factors highlights the importance of education on healthy lifestyles and proactive risk management to prevent acquired heart disease in ACHD patients.

## Supplementary Material

ehag216_Supplementary_Data

## Declarations

### Data Availability

Due to Swedish patient privacy law, we cannot make data available but interested researchers can contact us and we may help with presenting aggregated data.
