# Supplementary material for: Outcomes after a first acute myocardial infarction in patients with or without congenital heart disease
Source: Eur Heart J. 2026 May 11;47(29):3951–61. doi: 10.1093/eurheartj/ehag216 (PMC13429265; doi:10.1093/eurheartj/ehag216)
Supplement: ehag216_Supplementary_Data [file ehag216_supplementary_data.zip › Supplementary Figure 1 legends, 2026-01-27.docx]

**Supplementary Figure 1**. Flowchart showing the inclusion of all patients aged ≥18 years who had their first-time acute myocardial infarction (AMI) recorded in SWEDEHEART between 1 January 2000 and 2 February 2022. Data on patients with a diagnosis of congenital heart disease was retrieved from the Swedish National Registry of Congenital Heart Disease (SWEDCON). Patients with persisting foramen ovale (PFO), arrythmias, evaluations of physiological murmur and cardiomyopathies were excluded. * Patients with unknown admission time.
