# Supplementary material for: Outcomes after a first acute myocardial infarction in patients with or without congenital heart disease
Source: Eur Heart J. 2026 May 11;47(29):3951–61. doi: 10.1093/eurheartj/ehag216 (PMC13429265; doi:10.1093/eurheartj/ehag216)
Supplement: ehag216_Supplementary_Data [file ehag216_supplementary_data.zip › Supplementary Table1, 2026-01-27.docx]

**Supplemental Table 1: List of diagnosis codes in congenital heart disease**

CHD= adult congenital heart disease. ASD= atrial septal defect. AVSD= atrioventricular septal defect. EPCC= European Paediatric cardiac code. PDA= patent ductus arteriosus. VSD= ventricular septal defect.

| **Severe CHD** | **EPCC code** |
| --- | --- |
| CHD with pulmonary vascular disease (Eisenmenger syndrome) | 10.13.01, 10.13.02, 10.13.07, 10.13.08 |
| Any cyanotic CHD (unoperated or palliated) | 10.17.03 |
| Double-outlet ventricle | 01.01.04, 01.01.17, 01.01.18, 01.05.03, 12.27.01, 12.29.20  FHG00, FHG10, FHG96, FHH10 |
| Interrupted aortic arch | 09.15.03, 09.16.02, 09.29.16, 09.29.31  FDH00, FDH10, FDH96 |
| Pulmonary atresia (all forms) | 01.01.06, 01.01.07, 01.01.25, 09.05.11, 09.07.01, 09.07.11, 09.08.01  FHF00, FHF10, FHF20, FHF30, FHF00 |
| Transposition of the great arteries (except arterial switch) | 01.01.02, 01.01.03, 01.05.01  FDB00, FDB10, FDB20, FDB96, FFJ00, FFJ10, FFJ96 |
| Univentricular heart (including Fontan Circulation) | 01.01.09, 01.01.14, 01.01.22, 01.04.03, 01.04.04, 02.03.05, 06.01.00, 06.01.01, 06.02.01, 07.02.00, 07.07.00, 07.08.41, 07.08.42  FAE00, FAE10, FAE20, FAE30, FAE40, FAE50, FAF00, FAF10, FAF12, FAF20, FAF96, FBL40, FBL50, FDA00, FDA10, FDA96, FHH00 |
| Truncus arteriosus | 09.01.01, 09.01.02, 09.01.03, 09.02.00, 09.02.03, 09.04.01  FDC00, FDC10, FDC20, FDC96, FDD13, FDD20 |
| Other complex abnormalities (crisscross heart, ventricular inversion) | 02.01.02, 02.01.09, 02.03.03, 03.01.03 |

| **Moderate CHD** | **EPCC code** |
| --- | --- |
| Anomalous pulmonary venous connection (partial or total) | 01.01.16, 04.06.00, 04.07.01, 04.08.00, 04.08.05, 04.08.10, 04.08.20, 04.08.30, 04.08.91, 07.06.00  FFF00, FFF10, FFF20,  FFF96, FFG00, FFG20, FFG30, FFG96, FFH00, FFH10 |
| Anomalous coronary artery | 09.41.01, 09.45.01, 09.46.00, 09.46.01  FNJ00, FNJ12, FNK00, FNK96, FNW96 |
| Aortic stenosis - subvalvular or supravalvular | 07.09.00, 09.16.00  FDG00, FDG10, FDG96, FLE00, FLE10 |
| AVSD, partial or complete, including primum ASD | 06.06.00, 06.06.01, 06.06.08, 06.06.09. 06.07.26  FFD00, FFD20, FFD96, FHF96 |
| PDA, ASD secundum, moderate or large unrepaired (ventricular dysfunction or secondary pulmonary hypertension) | 10.16.08 OR 10.16.47 OR 10.13.20  PLUS  05.03.01, 05.03.02, 05.03.03, 05.04.01, 05.04.0205.04.03, 05.05.03, 09.27.00, 09.27.06, 09.27.24, 09.27.21, 14.10.41  OR  FFC00, FFC10, FFC22, FFC32, FFC50, FFC60, FFC96, FDE00, FDE10, FDE20, FDE31, FDE32, FDE96, FFL10 |
| Coarctation of the aorta | 09.28.00, 09.29.01, 09.29.02, 09.29.04, 09.29.05 09.29.08, 09.29.11  FCA50, FCC45, FDJ00, FDJ10, FDJ20, FDJ30, FDJ42, FDJ96 |
| Double chambered right ventricle | 07.03.01 |
| Ebstein anomaly | 06.01.34, 06.01.80, 06.02.23, 12.02.09  FGB00, FGB10, FGB96 |
| Peripheral pulmonary stenosis | 09.10.06 |
| Sinus of Valsalva aneurysm/fistula | 09.18.01, 09.18.03 |
| Tetralogy of Fallot | 01.01.01  FBL10, FBM10, FHE00, FHE10, FHE20, FHE30, FHE40, FHE96 |
| Transposition of the great arteries after arterial switch operation | FDB03 |
| VSD with associated abnormalities (ventricular dysfunction or secondary pulmonary hypertension) | 10.16.08 OR 10.16.47 OR 10.13.20  PLUS  07.10.00, 07.10.01, 07.11.01, 07.12.00, 07.14.02, 07.15.01, 07.15.04, 07.16.01, 07.20.00,  07.20.01  OR  FHB00, FHB10, FHB20, FHB30,  FHB40, FHB42, FHB50, FHB60,  FHB70, FHB80, FHB96, FHC00, FHC10, FHC20, FHC30, FHC96,  FHD00, FHD03, FHD10, FHD30, FHD96 |
| Parachute mitral valve, cleft leaflet | 06.02.36, 06.02.56 |
| Repaired aortic valve stenosis | FCA60, FMA00, FMA10, FMA20, FMA32, FMA96, FMC96, FMD00, FMD10, FMD12, FMD13, FMD96, FMW96, FMD20, FMD30, FMD33, FMD40 |

| **Mild CHD** | **EPCC code** |
| --- | --- |
| Congenital aortic valve disease and bicuspid aortic disease | 07.09.01, 09.15.00, 09.15.13, 09.15.22, 09.15.91, 09.15.92, 10.36.02, 10.36.04  FMC00, FMC10, FMC20 |
| Congenital mitral valve disease | 05.02.02, 06.02.00, 06.02.09, 06.02.35, 06.02.91, 06.02.92  FKA00, FKA10, FKA20, FKA32,  FKA96, FKB00, FKB10, FKB96, FKC00, FKC10, FKC20, FKC30, FKC40, FKC50, FKC60, FKC96, FKD00, FKD10, FKD20, FKD96, FKW96 |
| ASD, PDA or sinus venosus defect without associated abnormalities (ventricular dysfunction or secondary pulmonary hypertension) | 05.03.01, 05.03.02, 05.03.03, 05.04.01, 05.04.0205.04.03, 05.05.03, 09.27.00, 09.27.06, 09.27.24, 09.27.21, 14.10.41  FFC00, FFC10, FFC22, FFC32, FFC50, FFC60, FFC96, FDE00, FDE10, FDE20, FDE31, FDE32, FDE96, FFL10 |
| VSD without associated abnormalities (ventricular dysfunction or secondary pulmonary hypertension) | 07.10.00, 07.10.01, 07.11.01, 07.12.00, 07.14.02, 07.15.01, 07.15.04, 07.16.01, 07.20.00,  07.20.01  FHB00, FHB10, FHB20, FHB30,  FHB40, FHB42, FHB50, FHB60,  FHB70, FHB80, FHB96, FHC00, FHC10, FHC20, FHC30, FHC96,  FHD00, FHD03, FHD10, FHD30, FHD96 |
| Tricuspid valve abnormalities | 06.01.91, 06.01.92  FGA00, FGC00, FGC10, FGD00, FGD03, FGD10, FGD30, FGD40, FGD96, FGE00, FGE10, FGE20 FGE96, FGW96 |
| Pulmonary valve abnormalities | No diagnosis of Fallot PLUS  07.05.01, 07.05.30, 09.05.00, 09.05.01, 09.05.05, 09.05.27, 09.05.91, 09.05.92, 09.07.13, 09.09.01, 09.09.08, 09.10.00, 09.10.07, 09.10.11, 09.10.21  OR  FBA10, FBE00, FBE10 FBE20, FBE32, FBE35, FBE96, FBW96, FJE10, FJE20, FJF00, FJF10, FJF20, FJW96, FJD00, FJD10,  FJD20, FJE00, FJE30, FJE42, FJE96,  FJF12, FJF96 |
| Other CHD (cor triatriatum, coronary sinus abnormality, aorto — LV tunnel) | 04.04.00, 05.02.01, 09.17.02 |
