# Supplementary material for: Outcomes after a first acute myocardial infarction in patients with or without congenital heart disease
Source: Eur Heart J. 2026 May 11;47(29):3951–61. doi: 10.1093/eurheartj/ehag216 (PMC13429265; doi:10.1093/eurheartj/ehag216)
Supplement: ehag216_Supplementary_Data [file ehag216_supplementary_data.zip › Supplementary Table2, 2025-01-26.docx]

# Cox regression

Model 4

## Covariates Hazard Ratio (95% CI) p-value

**10-year Mortality**

| CHD vs control | 1.25 (0.85-1.84) | 0.253 |
| --- | --- | --- |
| Atrial fibrillation | 1.48 (1.00-2.19) | 0.047 |
| Aspirin | 0.47 (0.32-0.69) | 0.001 |
| Reduced systemic ventricular ejection fraction | 1.74 (1.54-1.97) | 0.001 |
| Current/ex-smoker | 1.47 (1.10-1.98) | 0.010 |
| Age | 1.06 (1.05-1.08) | 0.001 |
| Male sex | 1.00 (0.75-1.31) | 0.977 |
| Hypertension | 1.37 (1.05-1.80) | 0.021 |
| Type 2 diabetes mellitus | 1.57 (1.06-2.32) | 0.024 |
| Hypercholesterolemia | 0.59 (0.43-0.80) | 0.001 |
| Previous PCI | 1.03 (0.59-1.78) | 0.920 |
| Previous CABG | 2.02 (1.14-3.61) | 0.017 |
| **1-year Mortality** |  |  |
| CHD vs control | 0.52 (0.21-1.25) | 0.143 |
| Atrial fibrillation | 0.87 (0.39-1.91) | 0.724 |
| Aspirin | 0.15 (0.08-0.30) | 0.001 |
| Reduced systemic ventricular ejection fraction | 2.70 (2.08-3.50) | 0.001 |
| Current/ex-smoker | 2.29 (1.23-4.26) | 0.009 |
| Age | 1.05 (1.02-1.09) | 0.001 |
| Male sex | 1.36 (0.67-2.75) | 0.399 |
| Hypertension | 2.08 (1.04-4.16) | 0.040 |
| Type 2 diabetes mellitus | 0.91 (0.26-3.24) | 0.888 |
| Hypercholesterolemia | 0.41 (0.20-0.86) | 0.019 |
| Previous PCI | 1.18 (0.33-4.24) | 0.799 |
| Previous CABG | 2.85 0.68-11.89) | 0.151 |

**Supplementary Table 2.** Cox regression presenting hazard ratios (HRs) with 95% confidence intervals (CIs) for each individual covariate in relation to 1‑year and 10‑year mortality in patients with congenital heart disease (CHD) compared to matched controls based on age, sex, hypertension, diabetes, hyperlipidaemia, and history of percutaneous coronary intervention (PCI) or coronary artery bypass grafting (CABG). The model includes adjustment for the matching covariates age, sex, hypertension, diabetes, hypercholesterolaemia, PCI, and CABG, as well as additional clinical covariates: atrial fibrillation or flutter, aspirin use, systemic ventricular ejection fraction, and smoking status. The table illustrates the specific effect of each covariate on the fully adjusted model.
