# Supplementary figures and images for: Outcomes after a first acute myocardial infarction in patients with or without congenital heart disease
Source: Eur Heart J. 2026 May 11;47(29):3951–61. doi: 10.1093/eurheartj/ehag216 (PMC13429265; doi:10.1093/eurheartj/ehag216)

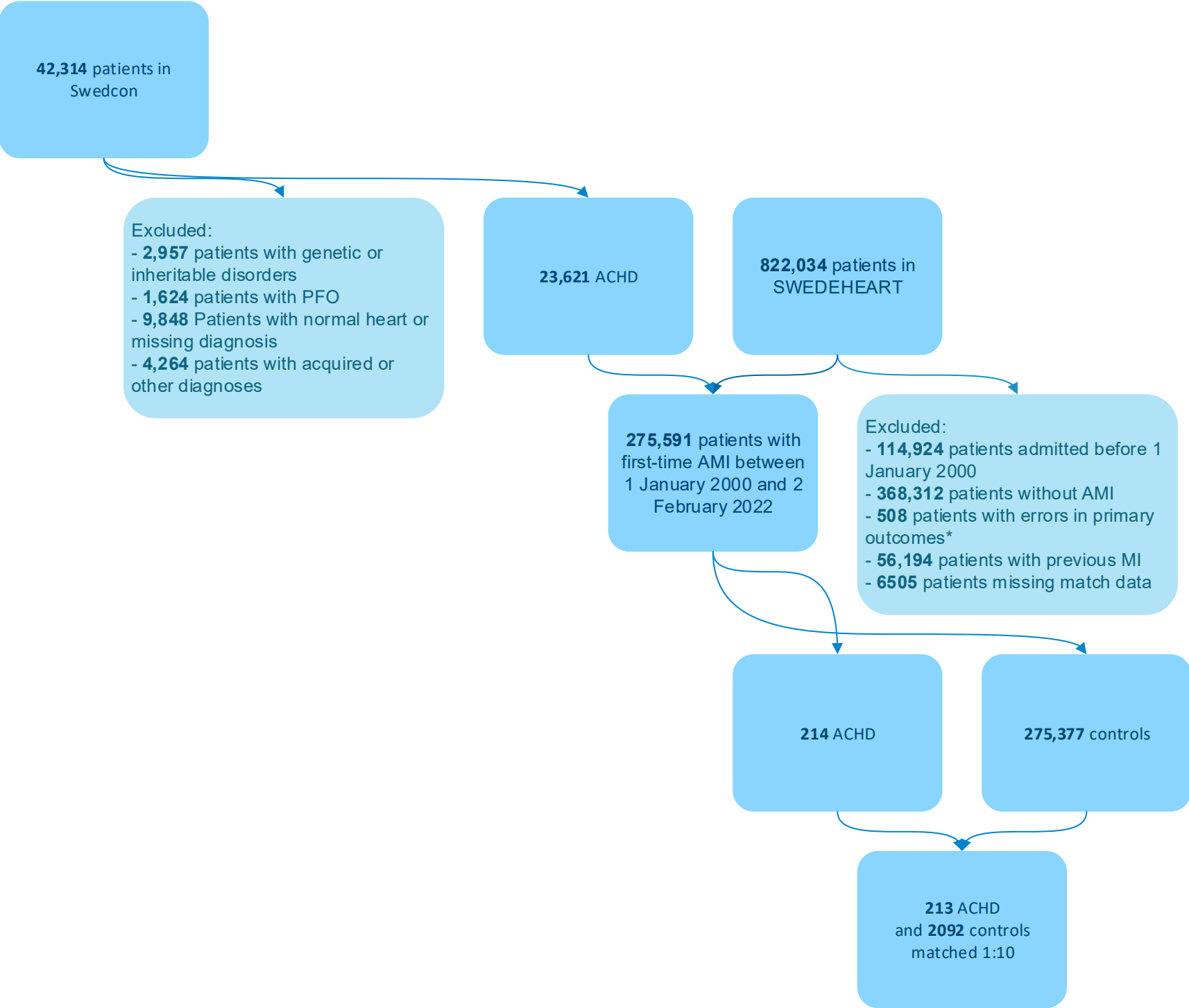

Supplement: ehag216_Supplementary_Data [file ehag216_supplementary_data.zip › Supplementary Figure 1.pdf]
